# Complementary Evaluation of Iron Deficiency Root Responses to Assess the Effectiveness of Different Iron Foliar Applications for Chlorosis Remediation

**DOI:** 10.3389/fpls.2018.00351

**Published:** 2018-03-20

**Authors:** Marta Fuentes, Eva Bacaicoa, Mikel Rivero, Ángel M. Zamarreño, José M. García-Mina

**Affiliations:** ^1^Biological and Agricultural Chemistry Group, Department of Environmental Biology, School of Sciences, University of Navarra, Pamplona, Spain; ^2^Iden Biotechnology, Cordovilla, Spain; ^3^Natural and Environmental Sciences Department, Public University of Navarra, Pamplona, Spain

**Keywords:** Fe plant nutrition, Fe deficiency, foliar Fe fertilization, Fe chelates, Fe(III)-chelate reductase, Fe (II) transporter, PM H^+^-ATPase, chlorosis

## Abstract

Iron deficiency in plants is caused by a low availability of iron in the soil, and its main visual symptom is leaf yellowing due to a decrease in chlorophyll content, along with a reduction in plant growth and fruit quality. Foliar sprays with Fe compounds are an economic alternative to the treatment with expensive synthetic Fe-chelates applied to the soil, although the efficacy of foliar treatments is rather limited. Generally, plant response to Fe-foliar treatments is monitored by measuring chlorophyll content (or related parameters as SPAD index). However, different studies have shown that foliar Fe sprays cause a local regreening and that translocation of the applied Fe within the plant is quite low. In this context, the aim of this study was to assess the effects of foliar applications of different Fe compounds [FeSO_4_, Fe(III)-EDTA, and Fe(III)-heptagluconate] on Fe-deficient cucumber plants, by studying the main physiological plant root responses to Fe deficiency [root Fe(III) chelate reductase (FCR) activity; acidification of the nutrient solution; and expression of the Fe deficiency responsive genes encoding FCR, *CsFRO1*, Fe(II) root transporter *CsIRT1*, and two plasma membrane H^+^-ATPases, *CsHA1* and *CsHA2*], along with SPAD index, plant growth and Fe content. The results showed that the overall assessment of Fe-deficiency root responses improved the evaluation of the efficacy of the Fe-foliar treatments compared to just monitoring SPAD indexes. Thus, FCR activity and expression of Fe-deficiency response genes, especially *CsFRO1* and *CsHA1*, preceded the trend of SPAD index and acted as indicators of whether the plant was sensing or not metabolically active Fe due to the treatments. Principal component analysis of the data also provided a graphical tool to evaluate the evolution of plant responses to foliar Fe treatments with time.

## Introduction

Iron deficiency is a nutritional disorder caused by a very low bioavailability of Fe in alkaline and/or calcareous soils. At this pH, Fe is predominantly in the form of precipitated Fe hydroxides. The ability of plants to mobilize Fe in those conditions is scarce, and the Fe deficiency status is revealed mainly as leaf yellowing (leaf chlorosis), but it also has a negative impact on plant growth and both fruit quality and yield (Lindsay and Schwab, 1982; Álvarez-Fernández et al., 2011).

According to the mechanisms developed to take up Fe from the rhizosphere when it is not readily available, plants have traditionally been divided into two groups (Marschner et al., 1986): Strategy I plants (dicotyledoneous and non-graminaceous monocots), which employ a Fe-reducing mechanism, and Strategy II plants (graminaceous monocots), which apply a chelation-based strategy, with the release of phytosiderophores with high affinity for ferric ions, whose complexes are then imported to the root.

Strategy I plants include the most relevant crops affected by Fe deficiency. When Fe bioavailability in the rhizosphere is low, proton plasma membrane ATPases located in the roots of Strategy I plants extrude protons to the rhizosphere in order to increase the solubility of Fe compounds. Although H^+^-ATPases are encoded by a multigene family, the acidification of the rhizosphere upon Fe-deficient conditions is due to specific root H^+^-ATPases, for example *AtHA2* in *Arabidopsis thaliana*, or *CsHA1* in *Cucumis sativus* (Santi et al., 2005; Santi and Schmidt, 2009). Once Fe has been solubilized, a membrane-bound ferric chelate reductase, encoded by the *FRO2* gene, reduces Fe(III) to Fe(II), which can be imported now by the high affinity iron uptake transporter, encoded by *IRT1* gene. This primary response to low Fe availability is integrated in a more complex regulation network (for a recent review, see Jeong et al., 2017 and references therein).

In order to overcome the problem of chlorosis related to low Fe availability in soils, commercial fertilizers containing Fe in different forms (inorganic salts, natural organic complexes, or synthetic chelates) may be applied, either as soil or foliar applications. Currently, the most efficient practice is the soil application of synthetic Fe chelates based on phenolic *N*-polycarboxylates, although the high cost of these compounds limits their use to the treatment of highly profitable crops (Abadía et al., 2004; Fernández et al., 2006). Besides, their high mobility and low biodegradability in soils poses the risk of contaminating natural ecosystems and water reservoirs. As an alternative, the use of foliar sprays containing soluble Fe is potentially less expensive since it normally involves inorganic Fe salts or cheaper natural Fe chelates (or complexes) (Fernández et al., 2006, 2013), although their efficacy is rather limited and full recovery from chlorosis is generally not achieved (Fernández et al., 2006; Rodríguez-Lucena et al., 2009, 2010; Pestana et al., 2012).

The assessment of the results derived from foliar application of Fe compounds is primarily performed by evaluating the regreening of the leaves, measuring SPAD values or chlorophyll content (El-Jendoubi et al., 2011). However, different studies have proven that this regreening only occurs in the areas where the treatment has been applied (El-Jendoubi et al., 2014) and that the mobility of the Fe inside the plant is rather limited, as also observed in studies tracing foliar ^59^Fe formulations (Nikolic et al., 2003; Rodríguez-Lucena et al., 2009). These experiments showed that the amount of iron that is taken up by the leaves depends on the iron compound foliarly applied and the plant species (among other factors), and that the portion of ^59^Fe translocated from the distal leaf tips, where the products have been applied, to other parts of the plant was around 5–25%. Nevertheless, less than 5% of the ^59^Fe that was taken up by chlorotic leaves was able to reach the basal part of the stem and the roots, irrespective of the iron compound applied or the plant species (Nikolic et al., 2003; Rodríguez-Lucena et al., 2009).

Whether this local action of foliarly applied Fe is able to trigger a systemic response that leads to the correction of Fe deficiency symptoms at a whole plant level is a question that cannot be answered by only measuring Fe concentration in plant organs and chlorophyll recovery in the leaves. In this context, the evaluation of the Fe-deficiency root responses in plants supplied with foliar Fe compounds may be a suitable strategy to better assess the real efficiency of the treatment. In this paper, we have investigated the efficacy of several Fe-foliar treatments to alleviate Fe deficiency in cucumber plants, by monitoring the variation of traditional parameters (SPAD index, Fe concentration in leaves and roots), along with the evolution of Strategy I root responses (root ferric chelate reductase activity and gene expression of two plasma membrane ATPases, *CsHA1* and *CsHA2*, the ferric chelate reductase *CsFRO1* and the Fe^2+^ transporter *CsIRT1*). Principal component analyses (PCA) of the data were performed in order to improve the consistency of the conclusions.

## Materials and Methods

### Plant Materials, Growth Conditions, and Treatments

Cucumber (*Cucumis sativus* L., cv. Ashley) seeds were germinated for a week in perlite moistened with 1 mM CaSO_4_ solution, at 24°C in darkness. Homogeneous seedlings were then selected and transferred to hydroponic receptacles placed in a growth chamber under the following conditions: 25/21°C day–night cycle, 70–75% relative humidity, and 15/9 h day–night photoperiod, 250 μmol m^-2^ s^-1^ photosynthetic photon flux density. Plants were cultivated for 11 days in an aerated nutrient solution containing: (in mM) 2 Ca(NO_3_)_2_, 0.75 K_2_SO_4_, 0.65 MgSO_4_, 0.5 KH_2_PO_4_, and (in μM) 50 KCl, 10 H_3_BO_3_, 1 MnSO_4_, 0.5 CuSO_4_, 0.5 ZnSO_4_ and 0.35 Na_2_MoO_4_, 5 Fe as Fe-EDDHA [Fe(III)-ethylene-diamine-dihydroxy-phenylacetic-acid, 85% otho-ortho isomer]. Nutrient solutions were renewed every 2–3 days and pH was set at 6.0.

Before the application of foliar treatments, nutrient solutions were renewed and plants were separated into two groups and supplied either with 40 μM Fe-EDDHA (+*Fe*: Fe resupply treatment), or with 0 μM Fe (for Fe-deficient control plants and foliar-treated plants).

We carried out two sets of experiments which differ in the treatments used, but whose plants were cultivated following the same protocol described above.

In the first set of experiments, after growing plants during 11 days as explained, the following treatments were imposed: (i) +*Fe*: 40 μM Fe-EDDHA resupply in the nutrient solution, no foliar Fe applied; (ii) -*Fe*: Fe-deficient plants, 0 μM Fe (no Fe supplied neither in the nutrient solution nor as a foliar application); (iii) *foliar 1X*: daily foliar application of 3 mM FeSO_4_, during 3 days (total amount of Fe applied: 2.51 mg Fe/plant); and (iv) *foliar 3X*: single foliar application of 9 mM FeSO_4_ (total amount of Fe applied: 2.51 mg Fe/plant).

In the second set of experiments, different forms of Fe were sprayed on leaves on a daily basis (three applications, total amount of Fe applied: 2.51 mg/plant). After growing plants in the conditions described above during 11 days, the following treatments were applied: (i) +*Fe*: 40 μM Fe-EDDHA resupply in the nutrient solution, no foliar Fe applied; (ii) -*Fe*: Fe-deficient plants, negative control, 0 μM Fe (no Fe supplied either in the nutrient solution nor as a foliar application); (iii) *FeSO_4_*: daily foliar application of 3 mM FeSO_4_, during 3 days; (iv) *Fe-HG*: daily foliar application of 3 mM Fe(III)-heptagluconate, during 3 days; and (v) *Fe-EDTA*: daily foliar application of 3 mM Fe(III)-ethylenediamine tetraacetic acid, during 3 days.

Iron sulfate and all iron chelates were of analytical grade. Foliar Fe treatments were always sprayed on the adaxial and abaxial surface of all leaves. The pH of the sprayed solutions was adjusted to 5.0 in order to avoid any effect on the ion-exchange properties of the cuticle (Fernández and Ebert, 2005). Potential Fe contamination of the nutrient solution was avoided by placing an aluminum sheet below the shoots and collecting potential drops coming from the leaves. All foliar treatments contained 0.1% of a surfactant (Biopower; sodic alkyl ether sulfate; Bayer Crop-Science), in order to assure a good distribution of sprayed solution on leaf surface. Previous studies showed this surfactant, used at 0.1%, did not affect Fe-root stress responses (data not shown). Control plants were also treated with a water solution of the surfactant at 0.1% and pH 5.0.

Plants were harvested 6, 24, 48, 72, and 96 h after Fe treatments. From the onset of the treatments, SPAD index and pH of the nutrient solutions was monitored. SPAD measurements were performed using a SPAD 502 m (Minolta, Co.) on the second and third leaves of same plants during the whole experiment. Values shown are means ± SE (*n* = 5 plants, 2 leaves per plant, 4 measurements per leave).

### Measurement of Fe(III)-Chelate Reductase Activity in Roots

The root FCR activity of cucumber plants was measured as in Pinton et al. (1999), using bathophenanthroline disulfonate (BPDS), which forms a red complex with Fe(II). One gram of excised apical roots from a single plant was incubated for 30 min in darkness at 25°C in 5.25 mL of nutrient solution (pH 5.5), containing 0.387 mM Fe(III)-EDTA and 0.286 mM BPDS. Immediately after the incubation, the formation of Fe(II)-BPDS complex was assessed by measuring the absorbance of the solution at 535 nm using an Agilent 8453 spectrophotometer with UV-visible Chemstation Software (Agilent Technologies, Santa Clara, CA, United States), using an extinction coefficient of 22.1 × 10^-3^ mM^-1^ cm^-1^. Previous assays showed a good correlation between these results and those obtained using intact plants (data not shown). Results shown are means ± SE of five independent activity measurements per treatment (FCR activity was individually measured in five roots excised from different plants).

### Total Fe Content in Roots

Total Fe content was analyzed after wet acidic digestion of five roots per treatment, individually processed as follows: roots were dried at 60°C and homogenized in a mill. 0.2 g of dried and powdered roots were digested with 6 mL HNO_3_ 65% and 2 mL H_2_O_2_ 33% in a microwave oven (Milestone-Ethos). Iron content was analyzed using ICP-OES spectrometry (Thermo Scientific; iCAB 6000 series).

### Real Time RT-PCR Analysis of mRNA Transcripts

Whole roots were harvested, immediately frozen in liquid nitrogen, and ground to a powder in a mortar with liquid nitrogen prior to RNA extraction. Five roots per treatment (from five different plants) were used to obtain five independent RNA biological replicates. Total RNA was extracted from 60 mg of powdered roots using the NucleoSpin RNA Plant Kit (Macherey-Nagel, Diirefn, Germany) following the manufacturer’s instructions, which included a digestion step with DNAse to eliminate genomic DNA contamination. The integrity and the concentration of the RNA in the extracts were assessed using the Experion Automated Electrophoresis System (Bio-Rad, Hercules, CA, United States) with RNA StdSens Chips. First strand cDNA synthesis was performed with the iScript cDNA synthesis kit (Bio-Rad, Hercules, CA, United States) in aliquots containing 1 μg RNA according to the manufacturer’s protocol.

Real time-PCR was carried out with 50 ng cDNA using iQ SYBR Green supermix containing hotstart iTaq DNA polymerase using an iCycler iQ (Bio-Rad Laboratories, Hercules, CA, United States). **Table 1** summarizes the primer pairs and the GenBank accessions for the genes studied in this paper: *CsHA1* and *CsHA2*, encoding two plasma membrane H^+^-ATPases (Santi et al., 2005); *CsFRO1*, encoding a Fe(III)-chelate reductase (Waters et al., 2007); *CsIRT1*, encoding the iron regulated Fe(II) transporter IRT1 (Waters et al., 2007); and two reference genes, *CsTUA* (α-tubulin; Santi et al., 2005) and *CsCYCLO* (cyclophilin; Cho et al., 2005). The reaction efficiency of PCR (between 1.99 and 2.02) was determined from the slope of standard dilution curves of pooled cDNAs with each specific primer pairs at harvest times.

**Table 1:** GenBank accession numbers and primer sequences used in the gene expression studies.

Gene	GenBank accession no.	Primer sequence	
*CsHA1*	AJ703810	Sense	5′-GGGATGGGCTGGTGTAGTTTG-3′
		Antisense	5′-GCTATCTCTGCTCGTCTCTTGG-3′
*CsHA2*	AJ703811	Sense	5′-TGAGCGACCTGGACTTCTATTG-3′
		Antisense	5′-GTGCCCATTGTGCTTCTCTTTC-3′
*CsIRT1*	AY590764	Sense	5′-TTCGCAGCAGGTATCATTCTCG-3′
		Antisense	5′-CACCACTCACTACAGGCAACTC-3′
*CsFRO1*	AY590765	Sense	5′-AGCGGCGGCAGTGGAATC-3′
		Antisense	5′-GTTTGGAGGAGGTGGAGGAAGG-3′
*CsCYCLO*	AY942800	Sense	5′-ATTTCCTATTTGCGTGTGTTGTT-3′
		Antisense	5′-GTAGCATAAACCATGACCCATAATA-3′
*CsTUA*	AJ715498	Sense	5′-ACCGTTGGAAAGGAAATTGTTG-3′
		Antisense	5′-GGAGCCGAGACCAGAACC-3′

Target gene expression was normalized to α-tubulin and cyclophilin expression. Relative expression (n-fold) of the normalized target gene in the treatments was calculated with the Relative Expression Software Tool-Multiple Condition Solver (REST-MCS beta 2.0 Software), via comparison to control plants considering efficiency values according to the mathematical model proposed by Pfaffl (2001) and Pfaffl et al. (2002). Significant differences in target gene expressions between treated and control plants were assessed by a pairwise fixed reallocation randomization test using the same software.

### Statistical Analysis

Significant differences (*P* ≤ 0.05) among treatments were calculated by using one-way analysis of variance (ANOVA) and the Fisher’s *post hoc* test. Statistical analysis of relative gene expression results was assessed using P-pairwise fixed reallocation randomization test.

Multivariate statistical analyses (PCA) were performed using the SPSS software version 12.0 (SPSS, Inc., Chicago, IL, United States).

## Results

### Single Versus Daily Foliar Application of FeSO_4_

The aim of the first set of experiments was to study the efficacy of two different ways of foliarly supplying the same total amount of Fe to chlorotic plants: either as a single application, or as a daily application of a more diluted solution of FeSO_4_. There were no significant differences in growth (fresh and dry weight of roots and shoots) or in total root Fe among treatments for the period of time of the experiment (data not shown).

Regarding SPAD values (**Table 2A**), starved control -*Fe* plants showed a decrease in SPAD index that was significant after 96 h from the onset of the foliar applications, and control +*Fe* plants (resupplied with Fe-EDDHA in nutrient solution) experienced an increase in SPAD values that was significant 48 h after the resupply. Both foliar treatments (*foliar 1X* and *foliar 3X*) caused a significant recovery of the SPAD index compared to control -*Fe* plants, that was higher in the case of the *foliar 3X* treatment although it did not reach the same SPAD levels as control +*Fe* plants.

**Table 2:** SPAD values measured in cucumber leaves from: **(A)** Experiment I and **(B)** Experiment II.

(A)	Time after treatments (hours)				

**Treatments**	**6 h**	**24 h**	**48 h**	**72 h**	**96 h**
-*Fe*	32,11 ± 4 cdefg	33,29 ± 5 bcde	33,36 ± 7 bcd	30,42 ± 6 fg	26,53 ± 6 h
+*Fe*	33,24 ± 5 bcde	35,30 ± 4 b	38,34 ± 5 a	40,90 ± 5 a	40,01 ± 5 a
*Foliar 1X*	31,33 ± 4 defg	31,52 ± 4 defg	32,39 ± 6 cdefg	33,20 ± 5 bcdef	30,08 ± 5 g
*Foliar 3X*	30,60 ± 3 efg	31,68 ± 3 defg	32,27 ± 4 cdefg	34,37 ± 4 bc	34,68 ± 4 bc

**(B)**	**Time after treatments (hours)**				

**Treatments**	**6 h**	**24 h**	**48 h**	**72 h**	**96 h**

-*Fe*	34,24 ± 4 b–i	33,37 ± 3 abcd	30,66 ± 3 f–j	30,55 ± 4 c–i	28,93 ± 4 ij
+*Fe*	35,13 ± 5 a–h	35,78 ± 5 a–f	36,60 ± 5 a–e	38,14 ± 4 a	38,16 ± 5 a
*FeSO_4_*	33,53 ± 5 d–j	37,10 ± 5 abc	35,07 ± 5 a–h	36,41 ± 4 a–e	37,42 ± 5 ab
*Fe-HG*	33,59 ± 4 d–j	35,73 ± 6 a–f	35,59 ± 5 a–g	35,95 ± 6 a–e	32,60 ± 4 f–j
*Fe-EDTA*	36,90 ± 5 e–j	32,82 ± 4 f-j	31,20 ± 4 hij	30,52 ± 5 j	32,28 ± 6 ghij

*Treatments: **(A)** -Fe, Fe-starved plants; +Fe, Fe-resupply in nutrient solution; Foliar 1X, foliar resupply with a single dose of 9 mM FeSO_4_ (2.54 mg Fe/plant); Foliar 3X, foliar resupply with three doses of 3 mM FeSO_4_ (2.54 mg Fe/plant). **(B)** -Fe, Fe-starved plants; +Fe, Fe-resupply in nutrient solution; FeSO_4_, foliar resupply with three doses of 3 mM FeSO_4_ (2.54 mg Fe/plant); Fe-HG, foliar resupply with three doses of 3 mM Fe as Fe-HG (2.54 mg Fe/plant); Fe-EDTA, foliar resupply with three doses of 3 mM Fe as Fe-EDTA (2.54 mg Fe/plant). Data are means ± SE (n = 5). Different letters indicate significant differences between treatments at P < 0.05 based on one-way ANOVA and Fisher’s post hoc test.*

**FIGURE 1:**
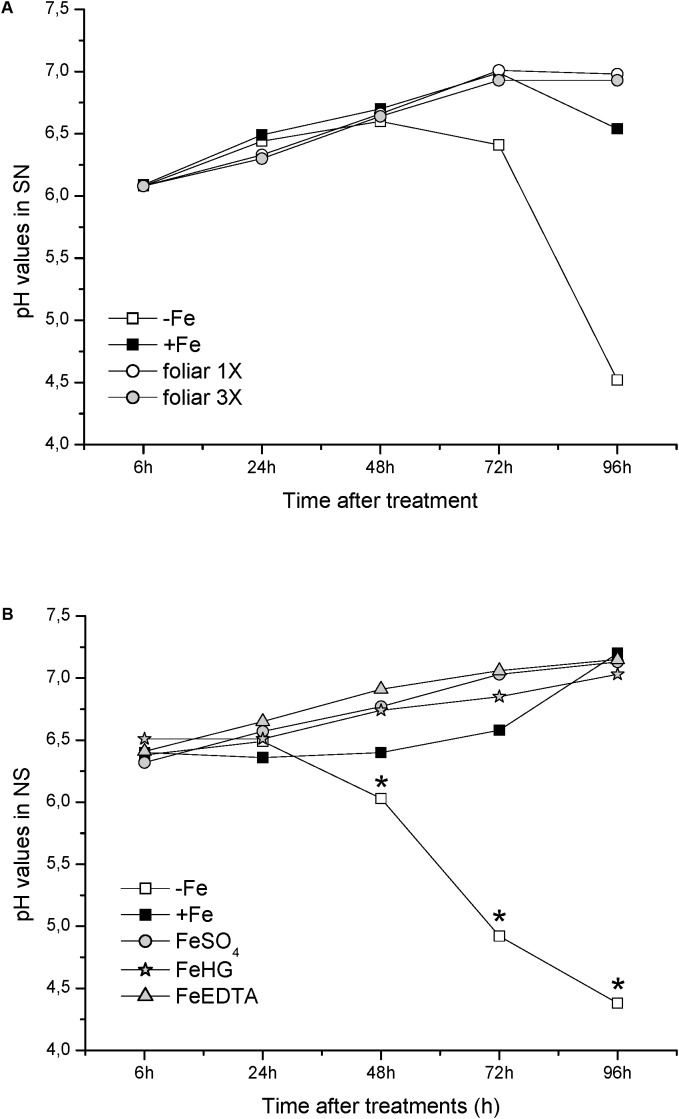
pH values measured in nutrient solution from: **(A)** Experiment I and **(B)** Experiment II. Data are means ± SE (*n* = 5). Asterisks indicate significant differences between treatments at *P* < 0.05 based on one-way ANOVA and Fisher’s *post hoc* test.

**FIGURE 2:**
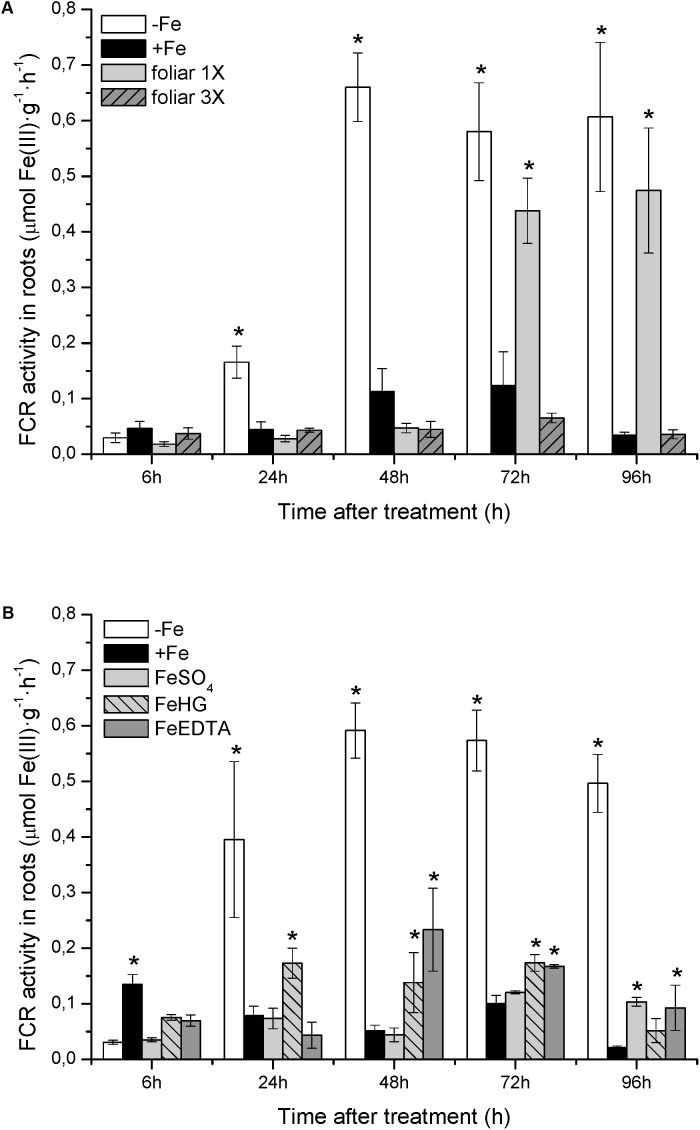
Fe(III)-chelate reductase (FCR) activity in cucumber roots measured from: **(A)** Experiment I and **(B)** Experiment II. Data are means ± SE (*n* = 5, from five independent roots). Asterisks indicate significant differences between treatments at *P* < 0.05 based on one-way ANOVA and Fisher’s *post hoc* test.

**FIGURE 3:**
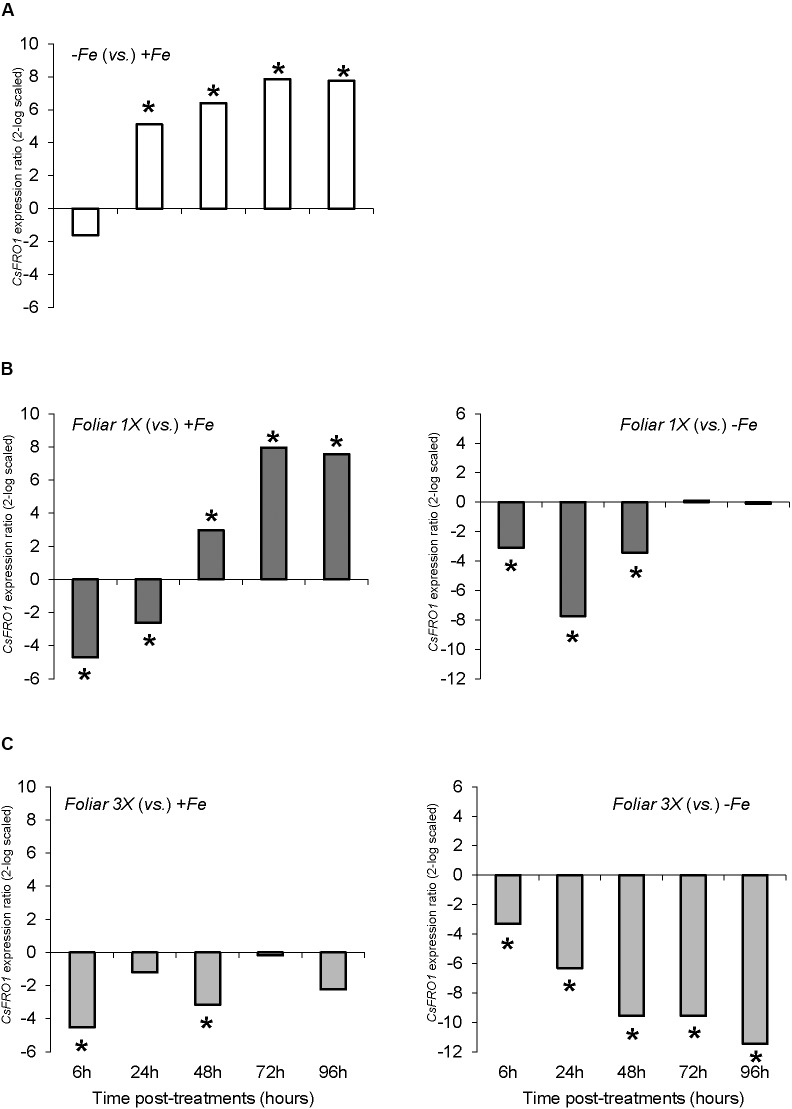
*CsFRO1* expression results in cucumber roots from Experiment I. Plants were grown in a nutrient solution with 5 μM Fe, and then some of them were transferred to nutrient solutions with 40 μM Fe (+*Fe*). The rest were transferred to nutrient solutions without Fe (–*Fe*). A third of the –*Fe* plants were sprayed with a single dose of 9 mM FeSO_4_ (2.54 mg Fe/plant, *Foliar 1X*), and other third was sprayed during 3 consecutive days with 3 mM FeSO_4_ (each plant received a total amount of 2.54 mg Fe/plant, *Foliar 3X*). **(A)** Expression of CsFRO1 in root of –*Fe* plants versus +*Fe* plants. **(B)** Expression of CsFRO1 in root of *Foliar 1X* plants versus +*Fe* (left) and –*Fe* (right) plants. **(C)** Expression of CsFRO1 in root of *Foliar 3X* plants versus +*Fe* (left) and –*Fe* (right) plants. Data are means ± SE (*n* = 5). Asterisks indicate significant differences between treatments at *P* < 0.05 based on one-way ANOVA and Fisher’s *post hoc* test. Statistical analysis of relative gene expression results was assessed using P-pairwise fixed reallocation randomization test.

With respect to physiological Fe-deficiency root responses, the results showed an acidification of the nutrient solution of Fe-starved plants (control -*Fe*) at 72 and 96 h compared to control +*Fe* plants, whereas the pH in the nutrient solution of *foliar 1X* and *foliar 3X* treated plants did not increase (**Figure 1A**). Control -*Fe* plants also increased the FCR activity in roots (**Figure 2A**) after 24 h compared with +*Fe* plants, with a more intense rise in activity at 48 h that was sustained at 72 and 96 h. The single application of foliar FeSO_4_ (*foliar 1X*) caused a transient effect, but FCR activity increased 72 and 96 h after the application of the treatment. Control +*Fe* and *foliar 3X* treated plants, on the other hand, did not show an enhanced root FCR activity. These results were in line with the gene expression of *CsFRO1* (**Figure 3**), whose expression was significantly increased in -*Fe* plant roots as compared with +*Fe* plants, from 24 to 96 h. *Foliar 1X* treatment caused a transient down-regulation of root *CsFRO1* with respect to +*Fe* plants (at 6 and 24 h), followed by a significant increase of the expression at 48, 72, and 96 h. The values observed at 72 and 96 h were not significantly different from those of -*Fe* at the same harvest times (**Figure 3**). On the other hand, *foliar 3X* caused a significant reduction in *CsFRO1* gene expression compared to -Fe plants throughout the experiment.

**FIGURE 4:**
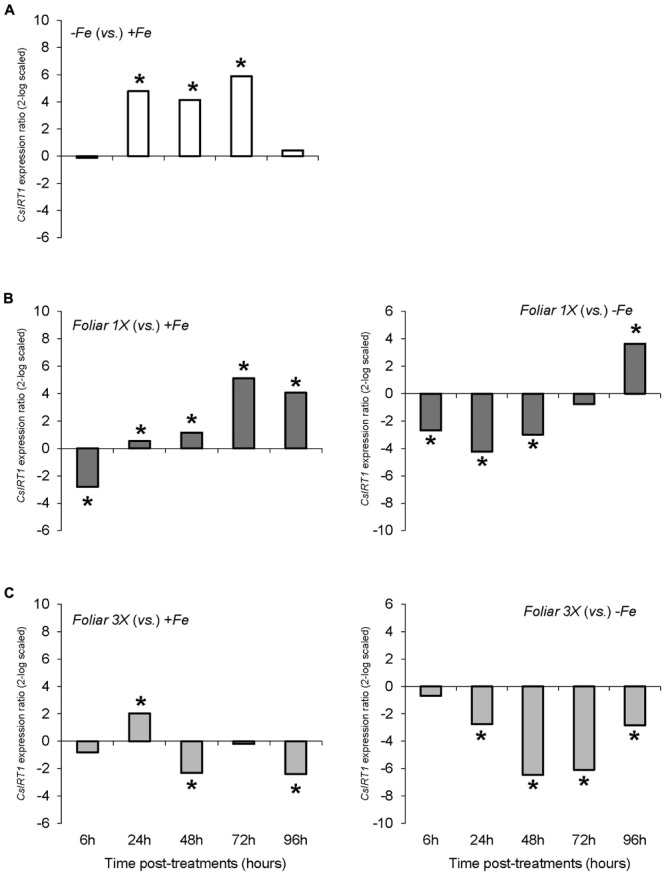
*CsIRT1* expression results in cucumber roots from Experiment I. Plants were grown in a nutrient solution with 5 μM Fe, and then some of them were transferred to nutrient solutions with 40 μM Fe (+*Fe*). The rest were transferred to nutrient solutions without Fe (–*Fe*). A third of the –*Fe* plants were sprayed with a single dose of 9 mM FeSO_4_ (2.54 mg Fe/plant, *Foliar 1X*), and other third was sprayed during 3 consecutive days with 3 mM FeSO_4_ (each plant received a total amount of 2.54 mg Fe/plant, *Foliar 3X*). **(A)** Expression of *CsIRT1* in root of –*Fe* plants versus +*Fe* plants. **(B)** Expression of *CsIRT1* in root of *Foliar 1X* plants versus +*Fe* (left) and –*Fe* (right) plants. **(C)** Expression of *CsIRT1* in root of *Foliar 3X* plants versus +*Fe* (left) and –*Fe* (right) plants. Data are means ± SE (*n* = 5). Asterisks indicate significant differences between treatments at *P* < 0.05 based on one-way ANOVA and Fisher’s *post hoc* test. Statistical analysis of relative gene expression results was assessed using P-pairwise fixed reallocation randomization test.

The results concerning the expression of the gene encoding the root Fe (II) transporter, *CsIRT1*, showed a different pattern than *CsFRO1* expression. Thus, Fe-starvation (-*Fe* plants) caused a significant increase in *CsIRT1* expression in comparison to +Fe at 24, 48, and 72 h (**Figure 4A**). The *foliar 1X* treatment was accompanied by a significant down-regulation of *CsIRT1* expression with respect to -*Fe* plants at 6, 24, and 48 h that disappeared at 72 h, whereas, when compared to +*Fe* plants, *foliar 1X* caused an up-regulation of *CsIRT1* at 24, 48, 72, and 96 h (**Figure 4B**). On the other hand, *foliar 3X* treatment was accompanied by a significant reduction in *CsIRT1* expression as compared with -*Fe* for 24 to 96 h (**Figure 4C**).

**FIGURE 5:**
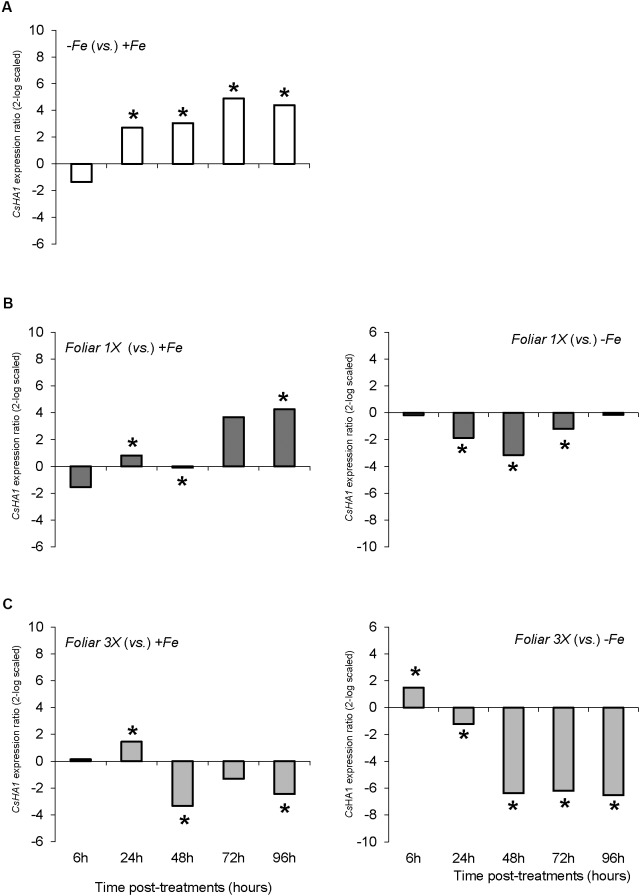
*CsHA1* expression results in cucumber roots from Experiment I. Plants were grown in a nutrient solution with 5 μM Fe, and then some of them were transferred to nutrient solutions with 40 μM Fe (+*Fe*). The rest were transferred to nutrient solutions without Fe (–*Fe*). A third of the –*Fe* plants were sprayed with a single dose of 9 mM FeSO_4_ (2.54 mg Fe/plant, *Foliar 1X*), and other third was sprayed during 3 consecutive days with 3 mM FeSO_4_ (each plant received a total amount of 2.54 mg Fe/plant, *Foliar 3X*). **(A)** Expression of *CsHA1* in root of –*Fe* plants versus +*Fe* plants. **(B**) Expression of *CsHA1* in root of *Foliar 1X* plants versus +*Fe* (left) and –*Fe* (right) plants. **(C)** Expression of *CsHA1* in root of *Foliar 3X* plants versus +*Fe* (left) and –*Fe* (right) plants. Data are means ± SE (*n* = 5). Asterisks indicate significant differences between treatments at *P* < 0.05 based on one-way ANOVA and Fisher’s *post hoc* test. Statistical analysis of relative gene expression results was assessed using P-pairwise fixed reallocation randomization test.

As regards the root plasma membrane H^+^-ATPases, *CsHA1* was significantly up-regulated from 24 to 96 h in Fe-deficient plants (-*Fe*) when compared with Fe-sufficient plants (+*Fe*) (**Figure 5A**). *Foliar 1X* treatment caused a transitory down-regulation of *CSHA1* with respect to -*Fe* plants, but these values were significantly higher than that of +*Fe* plants (**Figure 5B**), whereas *foliar 3X* reduced *CsHA1* expression in comparison with -*Fe* plants and showed less differences with +*Fe* plants (**Figure 5C**). No differences among treatments were observed for *CsHA2* expression (data not shown).

The results derived from each harvest were studied by PCA (**Figure 9**), generating two main factors (F1 and F2) explaining a high percentage of data variation (from 72% at 6 h to 91% at 96 h). Regarding the ability of PCA to discriminate among different treatments, the correlation of the score plots of the cases with F1 and F2 evolved with time (**Figure 9**, left), ending in a clear differential grouping of the treatments at 72 and 96 h. Thus F1 allowed the separation into two main groups (-*Fe* and *foliar 1X* versus +*Fe* and *foliar 3X*), while F2 discriminated each individual treatment. SPAD values and pH were highly correlated to F1 in the loading plot of 96 h, while *CsIRT1* showed a high correlation to F2 at this time (**Figure 9**, right). Additionally, the results for +*Fe* and *foliar 3X* evidenced a high correlation with pH and SPAD measurements at 96 h, whereas *foliar 1X* and -*Fe* plants were mainly correlated to Strategy I root responses.

### Efficiency of the Daily Foliar Application of Different Fe Compounds

No significant differences were observed in plant growth (fresh and dry weight of roots and shoots) or in total root Fe content between control (+*Fe* and -*Fe*) and treated plants (*FeSO_4_*, *Fe-HG*, *Fe-EDTA*) (data not shown). SPAD index, on the contrary, showed that *FeSO_4_* had a greater capacity for regreening the leaves than *Fe-HG* and *Fe-EDTA* (**Table 2B**).

Fe-starvation caused a significant and progressive acidification of the nutrient solution at 48, 72, and 96 h with respect to +*Fe* plants (**Figure 1B**). However, all Fe-foliar treatments (*FeSO_4_*, *Fe-HG*, and *Fe-EDTA*) avoided the acidification of the nutrient solution, presenting pH values similar to those of +*Fe* (**Figure 1B**).

The results of root FCR activity for +*Fe*, -*Fe*, and *FeSO_4_* plants were consistent with those observed in the previous study: (i) +*Fe* plants showed a low basal FCR activity; (ii) Fe-deficient plants (-*Fe*) enhanced root FCR activity at 24, 48, 72, and 96 h, (iii) roots of plants that received a daily application of FeSO_4_ (corresponding to *foliar 3X* treatment in the previous study) showed a low level of FCR activity, similar to that of +*Fe* plants (**Figure 2B**). *Fe-HG* and *Fe-EDTA* foliar treatments also caused a reduction in root FCR activity levels compared to -*Fe* plants, although they were slightly higher than those of +*Fe* and *FeSO_4_*.

**FIGURE 6:**
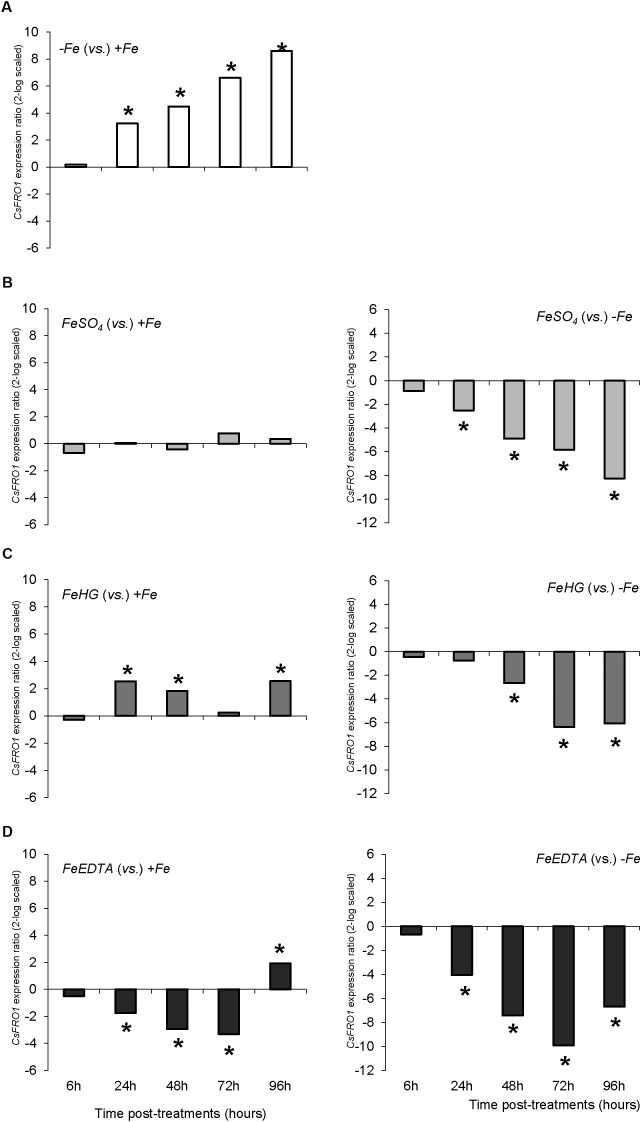
*CsFRO1* expression results in cucumber roots from Experiment II. Plants were grown in a nutrient solution with 5 μM Fe, and then some of them were transferred to nutrient solutions with 40 μM Fe (+*Fe*). The rest were transferred to nutrient solutions without Fe (–*Fe*). A fourth of the –*Fe* plants received no further treatments in order to function as a deficient control. The rest of the plants received a total amount of 2.54 mg Fe/plant (divided into a 3-day application) of one of the following treatments: *FeSO_4_*, 3 mM FeSO_4_; *Fe-HG*, 3 mM Fe(III)-heptagluconate; *Fe-EDTA*, 3 mM Fe(III)-EDTA. **(A)** Expression of *CsFRO1* in root of –*Fe* plants versus +*Fe* plants. **(B)** Expression of *CsFRO1* in root of *FeSO_4_* plants versus +*Fe* (left) and –*Fe* (right) plants. **(C)** Expression of *CsFRO1* in root of *Fe-HG* plants versus +*Fe* (left) and –*Fe* (right) plants. **(D)** Expression of *CsFRO1* in root of *Fe-EDTA* plants versus +*Fe* (left) and –*Fe* (right) plants. Data are means ± SE (*n* = 5). Asterisks indicate significant differences between treatments at *P* < 0.05 based on one-way ANOVA and Fisher’s *post hoc* test. Statistical analysis of relative gene expression results was assessed using P-pairwise fixed reallocation randomization test.

**FIGURE 7:**
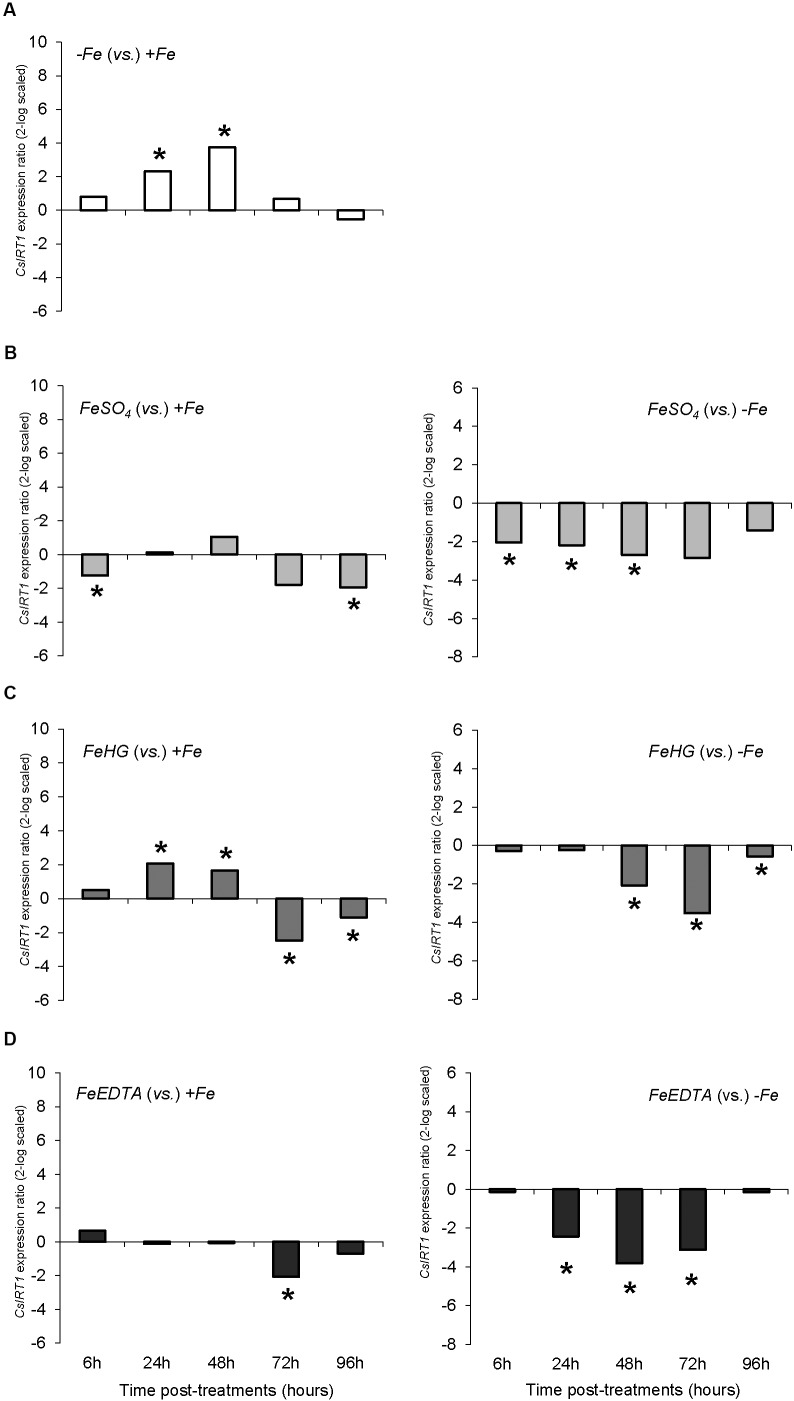
*CsIRT1* expression results in cucumber roots from Experiment II. Plants were grown in a nutrient solution with 5 μM Fe, and then some of them were transferred to nutrient solutions with 40 μM Fe (+*Fe*). The rest were transferred to nutrient solutions without Fe (–*Fe*). A fourth of the –*Fe* plants received no further treatments in order to function as a deficient control. The rest of the plants received a total amount of 2.54 mg Fe/plant (divided into a 3-day application) of one of the following treatments: *FeSO_4_*, 3 mM FeSO_4_; *Fe-HG*, 3 mM Fe(III)-heptagluconate; *Fe-EDTA*, 3 mM Fe(III)-EDTA. **(A)** Expression of *CsIRT1* in root of –*Fe* plants versus +*Fe* plants. **(B)** Expression of *CsIRT1* in root of *FeSO_4_* plants versus +*Fe* (left) and –*Fe* (right) plants. **(C)** Expression of *CsIRT1* in root of *Fe-HG* plants versus +*Fe* (left) and –*Fe* (right) plants. **(D)** Expression of *CsIRT1* in root of *Fe-EDTA* plants versus +*Fe* (left) and –*Fe* (right) plants. Data are means ± SE (*n* = 5). Asterisks indicate significant differences between treatments at *P* < 0.05 based on one-way ANOVA and Fisher’s *post hoc* test. Statistical analysis of relative gene expression results was assessed using P-pairwise fixed reallocation randomization test.

**FIGURE 8:**
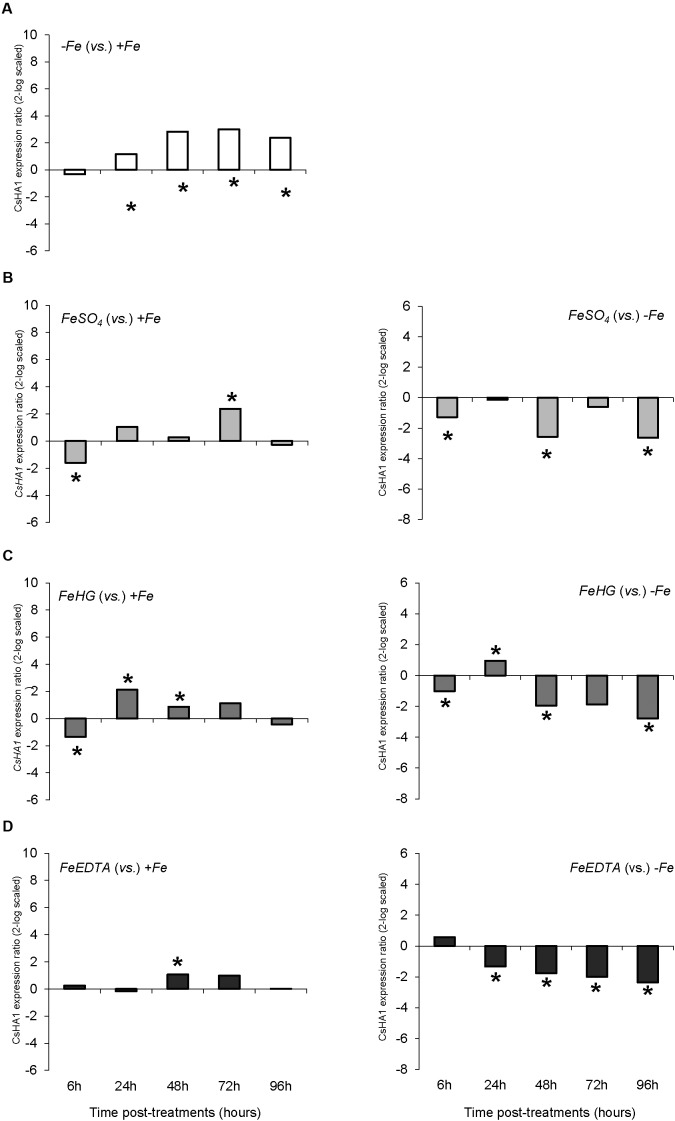
*CsHA1* expression results in cucumber roots from Experiment II. Plants were grown in a nutrient solution with 5 μM Fe, and then some of them were transferred to nutrient solutions with 40 μM Fe (+*Fe*). The rest were transferred to nutrient solutions without Fe (–*Fe*). A fourth of the –*Fe* plants received no further treatments in order to function as a deficient control. The rest of the plants received a total amount of 2.54 mg Fe/plant (divided into a 3-day application) of one of the following treatments: *FeSO_4_*, 3 mM FeSO_4_; *Fe-HG*, 3 mM Fe(III)-heptagluconate; *Fe-EDTA*, 3 mM Fe(III)-EDTA. **(A)** Expression of *CsHA1* in root of –*Fe* plants versus +*Fe* plants. **(B)** Expression of *CsHA1* in root of *FeSO_4_* plants versus +*Fe* (left) and –*Fe* (right) plants. **(C)** Expression of *CsHA1* in root of *Fe-HG* plants versus +*Fe* (left) and –*Fe* (right) plants. **(D)** Expression of *CsHA1* in root of *Fe-EDTA* plants versus +*Fe* (left) and –*Fe* (right) plants. Data are means ± SE (*n* = 5). Asterisks indicate significant differences between treatments at *P* < 0.05 based on one-way ANOVA and Fisher’s *post hoc* test. Statistical analysis of relative gene expression results was assessed using P-pairwise fixed reallocation randomization test.

The variations in *CsFRO1* expression profiles were similar to those of root FCR activity, with some differences depending on the treatment (**Figure 6**). All the plants that received a resupply of iron (+*Fe* and foliar treatments) showed down-regulated *CsFRO1* expression compared to -*Fe* plants. On the other hand, only *Fe-HG* plants showed up-regulated *CsFRO1* expression compared to +*Fe* plants. A similar trend was observed in the results concerning *CsIRT1* (**Figure 7**). With regard to the expression of *CsHA1* (**Figure 8**), -*Fe* plants presented a significant increase with respect to +*Fe* plants at 24, 48, 72, and 96 h (**Figure 8A**). No significant changes were observed for *CsHA2* expression between controls and treatments (data not shown). *FeSO_4_* significantly up-regulated *CsHA1* expression with respect to -*Fe* at 6, 48, and 96 h (**Figure 6B**). The reduction in *CsHA1* expression at 72 h was not significant versus -*Fe*, but significantly higher than that of +*Fe* (**Figure 8B**). *Fe-HG* caused a significant down-regulation of *CsHA1* in comparison with -*Fe* at 6, 48, and 96 h (**Figure 8C**), whereas *Fe-EDTA* down-regulated *CsHA1* expression from 24 to 96 h compared with -*Fe* (**Figure 8D**).

**FIGURE 9:**
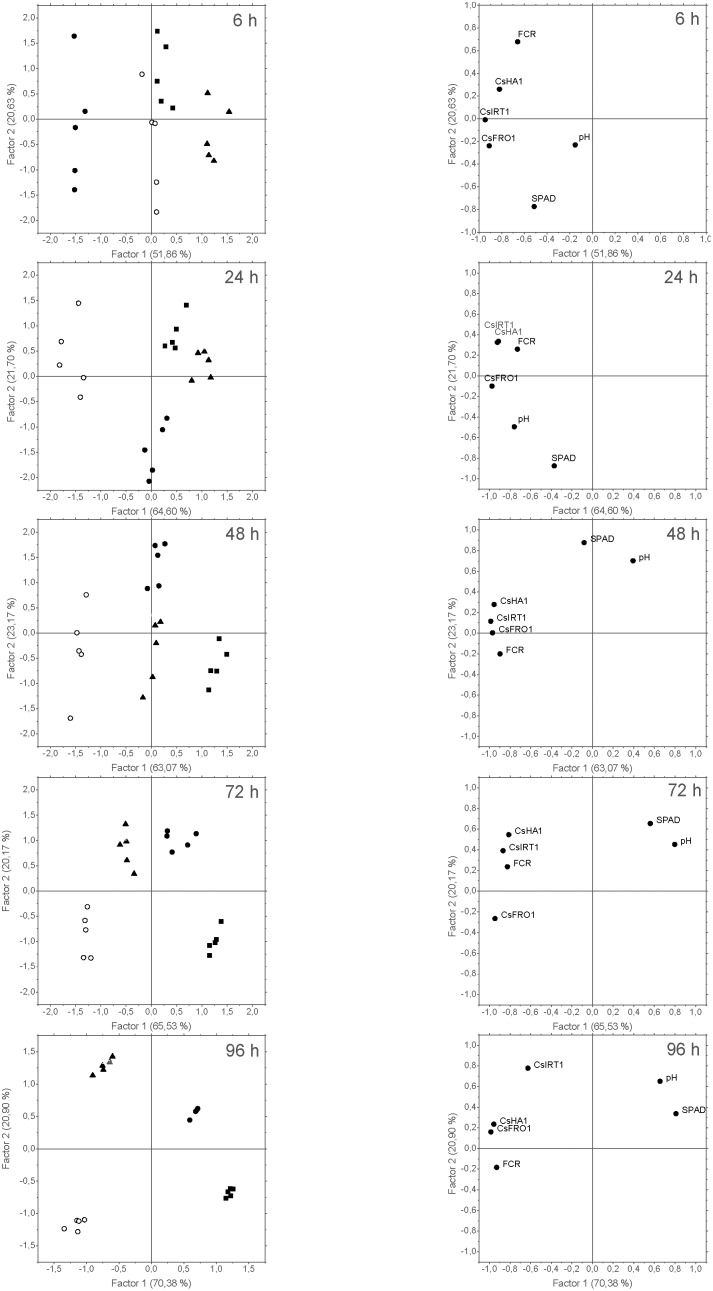
>Score plots of the cases **(left)** and loading plots of the variables **(right)** from the PCA of data derived from Experiment I. Five cases per treatment were considered: +Fe control (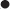), –Fe control (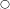), 3 mM FeSO_4_ (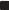), and 9 mM FeSO_4_ (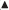). The variables introduced in the analysis were: SPAD values measured in leaves (SPAD); pH values measured in NS (pH); *CsFRO1*, *CsIRT1*, and *CsHA1* relative expression results (CsFRO1, CsIRT1, CsHA1); and FCR activity in roots (FCR). The number in brackets is the percentage of total variance explained by the factor.

**FIGURE 10:**
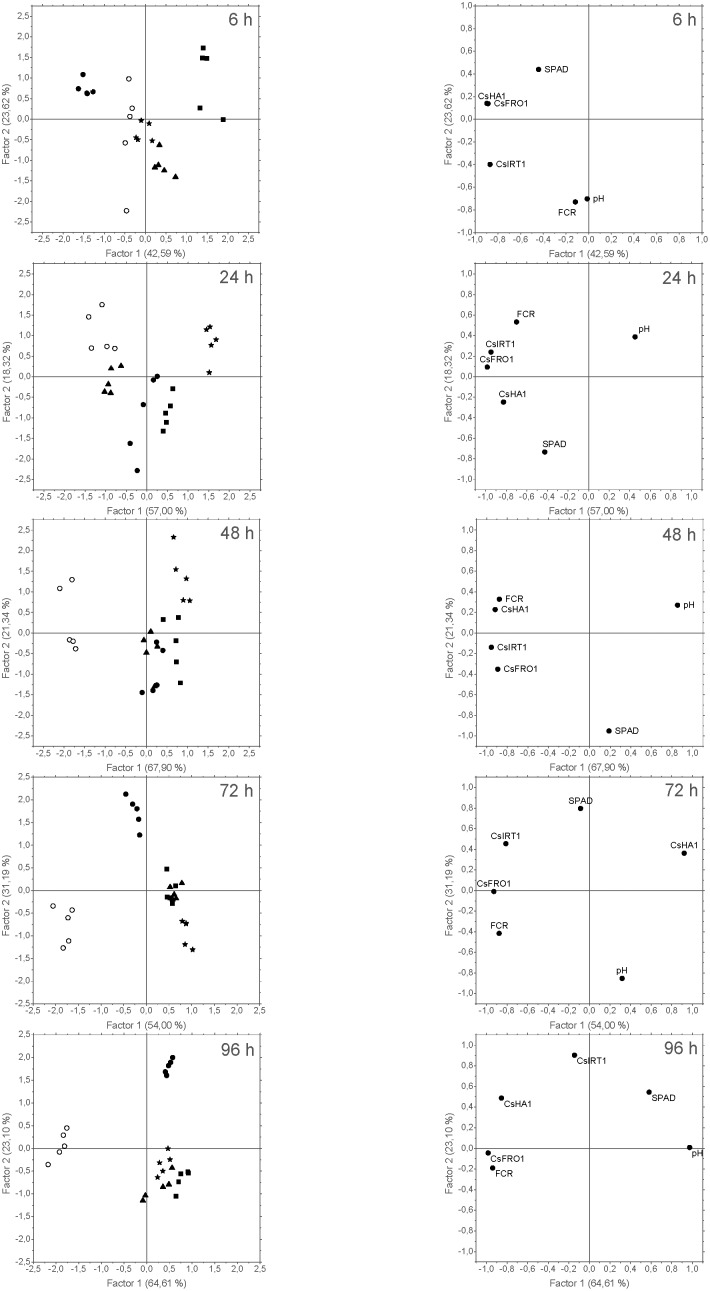
Score plots of the cases **(left)** and loading plots of the variables **(right)** from the PCA of data derived from Experiment II. Five cases per treatment were considered: +Fe control (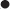), –Fe control (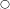), FeSO_4_ (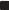), HG (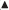), and EDTA (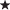). The variables introduced in the analysis were: SPAD values measured in leaves (SPAD); pH values measured in NS (pH); *CsFRO1*, *CsIRT1*, and *CsHA1* relative expression results (CsFRO1, CsIRT1, CsHA1); and FCR activity in roots (FCR). The number in brackets is the percentage of total variance explained by the factor.

The results related to PCA of data from Experiment II showed trends that were similar to those found in the previous experiment, with two main factors (F1 and F2) explaining 66% (at 6 h) to 87% (96 h) of the variance. Concerning PCA grouping efficacy for the different treatments, the score plots for the cases (**Figure 10**, left) showed that F1 tended to separated chlorotic (-*Fe*) from Fe-resupplied (+*Fe*, *Fe-EDTA*, *FeSO_4_*, *Fe-HG*) plants, while F2 discriminated between +Fe and foliar Fe treatments (*FeSO_4_*, *Fe-EDTA*, and Fe-HG). As it was also observed in the previous experiments, after 96 h SPAD and pH values and Fe-deficiency root responses were opposed in the loading plots (**Figures 9**, **10**, right).

## Discussion

Many studies have shown that the development of specific root responses to Fe-starvation is closely controlled by the presence of Fe-physiological or metabolic deficiency in the shoot (Vert et al., 2003; Enomoto et al., 2007; García et al., 2013; and reviews by Hindt and Guerinot, 2012; Ivanov et al., 2012 and references therein). Therefore, the evaluation of these root responses to Fe-deficient conditions might be a complementary tool to assess the efficacy of Fe-foliar treatments, besides traditional SPAD monitoring.

When we analyze the results concerning shoot-related parameters (shoot dry matter, SPAD values) and root-related parameters (root dry matter, FCR activity, nutrient solution acidification, *CsFRO1*, *CsIRT1*, and *CsHA1* expression) for +*Fe* and -*Fe* treatments, we observe that while there were no differences in growth and the differences in SPAD were only significant at 72 and 92 h (as in the case of nutrient solution acidification), the increase in root FCR activity as well as the *CsFRO1*, *CsIRT1*, and *CsHA1* gene expression associated with Fe-starvation was significant from 24 h (**Figures 1A**, **2**, **3A**, **4**, **5** and **Table 2A**). This fact, which has been reported in other studies, shows that the sensitiveness (faster response) to plant Fe-deficiency evolvement is quite different between the parameters normally measured in the shoot (principally, SPAD or chlorophyll content) and those corresponding to the main Fe-deficiency root responses (Bacaicoa and García-Mina, 2009; Pestana et al., 2012; García et al., 2013).

In the evaluation of *foliar 1X* and *foliar 3X* treatments, changes in the expression levels of Strategy I root response genes and in the root FCR activity preceded the trend of SPAD values. Thus, in *foliar 1X*, roots sensed a deficiency of metabolically active Fe in shoots and activated root responses to Fe-starvation at 48 h, before the SPAD index started to decrease. In the case of *foliar 3x*, although it was not able to regreen the leaves to the same levels as in +*Fe*, the measured root responses indicated that this treatment was able to deactivate the Fe-deficiency machinery, and therefore that the repeated foliar application of FeSO_4_ was being effective although at a slow rate, as shown by the SPAD values. In order to obtain a sustained alleviation of Fe-deficiency, it is more efficient to perform several and less concentrated Fe foliar applications than a single one. This may be because the repeated application reduces potential losses due to Fe precipitation in leaf-apoplast and due to leaching from leaf surface (Fernández and Brown, 2013; Fernández et al., 2013; García-Mina et al., 2013; El-Jendoubi et al., 2014).

Principal component analysis of these data taken altogether has also proven to be a useful tool to assess the overall response of the plant to the treatments. The pattern of the score plots of the cases evolved with time, and these plots display the similarity in the response to the treatments, allowing us to evaluate if the treatment is being effective. Thus, time-course pattern of PCA score plots shows that response to *foliar 1X* treatments (according to the measured parameters) is progressively approaching that of +*Fe* plants that received a resupply of completely available iron in the form of Fe-EDDHA in the nutrient solution.

Once determined that the best way to proceed with the foliar treatments was to apply a daily dose of the Fe compound, a second set of experiments with different Fe compounds (FeSO_4_, Fe-EDTA, and Fe-HG) was performed. According to SPAD index values, FeSO_4_ was the most effective foliar treatment to alleviate iron chlorosis, at least under our experimental conditions. On the contrary, the gene expression evaluation showed that all the treatments significantly down-regulated Fe-deficiency response genes compared to -*Fe* plants. This may indicate that the foliar treatments are being effective in delivering “active” iron, but either the recovery of SPAD is very slow, or the amount of delivered Fe that can be metabolically useful is scarce.

The differences among treatments could be ascribed to the fact that the rate of Fe uptake in leaves depends on the Fe compound that has been supplied. Firstly, photochemical reduction of the Fe depends on the chemical species to which Fe is bound. Thus, for example, Larbi et al. (2001) presented data regarding photoreduction of Fe(III)-EDTA, Fe(III)-citrate and Fe(III)-malate, among which ferric citrate was by far the most affected by light photochemical reduction, although this process was not negligible for Fe-EDTA (or Fe-malate as well). Secondly, studies with labeled ^59^Fe compounds have shown that the amount of foliarly applied Fe that is taken up by plants is different depending on the Fe compound. In this way, Nikolic et al. (2003) and Rodríguez-Lucena et al. (2009) reported that leaves were able to take up ^59^Fe from ^59^Fe-EDTA in a greater extent that from ^59^Fe-humic or ^59^Fe-lignosulfonate complexes. However, the same studies also showed that the amount of ^59^Fe translocated to other parts of the plants, and specifically to the root, was surprisingly similar despite the differences in ^59^Fe uptake, what might be one reason why there were no differences in iron root content among treated and non-treated plants. Lastly, Zamboni et al. (2016) in a transcriptomic study in Fe-deficient tomato plants recently found that the root transcriptional response to the Fe resupply in the nutrient solution depends on the nature of the chelating agent.

## Conclusion

The results presented here indicate that the complementary evaluation of the main physiological Fe deficiency root responses along with chlorophyll content-related parameters (as SPAD index) is a useful tool to better assess the ability of Fe-foliar sprays to remedy Fe-deficiency in rapid screening studies carried out at laboratory level. Regarding the relative relevance of all the parameters that have been analyzed, root FCR activity and relative expression of *CsFRO1* and *CsHA1* have proven to be the most informative ones in cucumber, in addition to SPAD index. PCA also helped in assessing the evolution of the plant response to treatments with time.

## Author Contributions

MF, EB, and JG-M designed the experiments. MR, MF, and EB made the experiments. MF made the transcriptomic study. MR, MF, EB, ÁZ, and JG-M discussed and interpreted the results. JG-M and MF wrote the MS.

## Conflict of Interest Statement

The authors declare that the research was conducted in the absence of any commercial or financial relationships that could be construed as a potential conflict of interest.
